# Treatment Outcome of Tuberculosis and Associated Factors among TB-HIV Co-Infected Patients at Public Hospitals of Harar Town, Eastern Ethiopia. A five-year retrospective study

**DOI:** 10.1186/s12889-019-7980-x

**Published:** 2019-12-10

**Authors:** Assefa Tola, Kirubel Minsamo Mishore, Yohanes Ayele, Abraham Nigussie Mekuria, Nanati Legese

**Affiliations:** 1 Department of Epidemiology and Biostatistics, School of public health, College of Health and Medical Sciences, Haramaya University, Harar, Ethiopia; 2 Department of Clinical Pharmacy, School of Pharmacy, College of Health and Medical Sciences, Haramaya University, Harar, Ethiopia; 3 Department of Pharmacology, School of Pharmacy, College of Health and Medical Sciences, Haramaya University, Harar, Ethiopia; 4 Department of pharmaceutics and social pharmacy, School of Pharmacy, College of Health and Medical Sciences, Haramaya University, Harar, Ethiopia

**Keywords:** TB- HIV co-infected, TB treatment outcome, Harar

## Abstract

**Background:**

The bidirectional relationship between the twin epidemics of Tuberculosis (TB) and Human Immunodeficiency Virus (HIV) causes major global health challenges in the twenty-first century. TB-HIV co-infected people are facing multifaceted problems like high lost to follow up rates, poor treatment adherence, high TB recurrence rate, and high mortality risk. Our objective was to a*ssess the outcomes* of TB *treatment and associated factors among TB-HIV co-infected patients in Harar town, Eastern part of Ethiopia*, 2018.

**Methods:**

A retrospective study was conducted among systematically selected 349 TB/HIV co-infected patients who registered from 2012 to 2017 in two public hospitals in Harar town. The data were collected through document review by using a pre-tested structured data extraction checklist. The data were analyzed using SPSS Version 21. Bivariate and multivariate logistic regression were determined at 95% confidence intervals.

**Results:**

Among the 349 TB/HIV co-infected patients included in the study, 30.1% were cured, 56.7% had completed their treatment, 7.7% died, 1.7% were lost to follow up, and 3.7% were treatment failure. Overall, 86.8% of the TB-HIV co-infected patients had successful TB treatment outcomes. The patients who were on re-treatment category (AOR = 2.91, 95% CI: 1.17–7.28), who had a history of opportunistic infection (AOR = 3.68, 95% CI: 1.62–8.33), and who did not take co-trimoxazole prophylaxis (AOR = 3.54, 95% CI: 1.59–7.89) had 2.91, 3.68, and 3.54 times higher odds of having unsuccessful TB treatment outcome than their counterparties, respectively. The chance of unsuccessful TB treatment outcome was 4.46 (95% CI: 1.24–16.02), 5.94 (95% CI: 1.87–18.85), and 3.01 (95% CI: 1.15–7.91) times higher among TB/HIV patients in stage 2, 3 and 4 than those in stage 1, respectively.

**Conclusions:**

The overall rate of the success of the TB treatment among TB-HIV co-infected patients in this study was higher compared with many previous studies. TB/HIV patients with a history of previous TB treatment, smear-positive pulmonary TB, late HIV stage, history of opportunistic infection and not being on co-trimoxazole prophylaxis therapy were at a high risk of getting poor treatment outcomes.

## Background

The twin epidemics of Tuberculosis (TB) and Human Immunodeficiency Virus (HIV) are the major global health challenges of the twenty-first century [1, 2]. These two infectious diseases have a bidirectional relationship that poses a dual public health burden to resource-limited countries [2]. TB-HIV co-infected people are experiencing “double trouble” that puts them at high risk of mortality, rapid disease progression, and development of other opportunistic infection [3, 4].

HIV, because of its immunosuppressive nature, is a strong risk factor for the development of TB [5] and reactivation of latent TB [4, 6]. In 2017, globally about 920,000 people who were infected with HIV developed TB [7]. Reports also indicate that people living with HIV are 20 to 21times more likely to develop active TB than those HIV negative people [7, 8]. On the other hand, TB is the most frequent life-threatening opportunistic infection and the leading cause of death among HIV positive people [1, 3, 6–9]. About one in four deaths among HIV positive people is caused by TB [3]. TB speeds up the viral replication and load in HIV infected people [4, 6]. In general, TB affects about one-third of the 36.7 million people living with HIV worldwide [10].

Ethiopia is one of the 14 countries with high-burden of all TB, TB/HIV, and Multi-drug Resistant TB (MDR-TB) in the world [5]. According to studies and reports, the prevalence of TB-HIV co-infection in the country is so high that it has ranged from 6.1 to 40.4% [2, 4, 5, 11, 12]. The global 2018 TB report showed that in Ethiopia an estimated 3600 (95% CI: 2500–5000) TB-HIV co-infected people died while on TB treatment in the year 2017 alone [5].

In TB-HIV co-infected patients, HIV affects the effectiveness and success of TB treatment in many ways [5]. For example, the co-infected patients are exposed to many regimens including Antiretroviral Therapy (ART), anti-TB therapy, and preventive therapy of HIV-related co-morbidities which in turn is associated with an increased incidence of adverse drug reaction, poor adherence often due to pill burden, and decreased drug effectiveness. Consequently, the patient may experience a high default rate leading to TB recurrence and increased risk of death [4, 13]. Hence, well-coordinated therapeutic management is needed to ensure optimum treatment outcomes in terms of response and prevention of drug resistance.

Collaborative TB/HIV activities and management of comorbidities are the key components of the ‘end TB strategy’ [5, 13, 14]. To reduce the dual burden of TB/HIV among people living with HIV, it is recommended to scale up the three I’s, which are intensified TB case-finding followed by high-quality TB treatment, isoniazid preventive therapy (IPT), and infection control for TB in all congregate settings and health facilities providing HIV care [6, 9, 10].

According to Ethiopia national guidelines, all HIV positive patients should be evaluated for TB before ART is initiated and then at every visit. Similarly, all TB patients should be offered HIV testing services in TB clinics [4, 6, 15]. Regarding the therapeutic management, ART should be provided to all TB/HIV co-infected patients regardless of CD4 count or WHO stage. However, ant-tuberculosis treatment should be provided first, followed by ART within the first 8 weeks of treatment [6, 15]. Likewise, Co-trimoxazole prophylaxis (CPT) should be provided to all TB-HIV co-infected patients, regardless of their CD4 count [4, 6, 9, 15]. In order to prevent the reactivation of latent TB, after excluding the presence of active TB infection, IPT should be given to all people with HIV at least for six months [6, 10].

The percentage of patients treated successfully is a key indicator for monitoring and evaluating the effectiveness of the TB Directly Observed Therapy (DOT) program [10]. This is particularly necessary for patients with TB-HIV co-morbidities where the treatment outcome could be affected by many factors. Hence, it is important to conduct a periodic evaluation of the treatment outcome for this segment of the population to assess the level of quality of care and to imply possible directions for improvement. Unfortunately, there are only a few studies that have assessed TB treatment outcomes among TB-HIV co-infected people in Eastern Ethiopia. Hence, our objective was to assess the outcomes of TB treatment and the *associated factors* among TB-HIV co-infected patients in public hospitals in Harar town, Eastern Ethiopia.

## Methods

### Study setting

This study was done in Harar town, which is the capital town of Harari Regional State. In the town, there are five hospitals: Hiwot Fana Specialized University Hospital (HFSUH), Jugal Hospital, Yimaj Hospital, Harar General Hospital, and Federal Police Hospital. Only the first two are public hospitals.

### Study design and period

An institution-based retrospective study was conducted from April 1–10, 2018 to determine TB *treatment outcome and the associated factors among the TB-HIV co-infected patients in the two public hospitals in Harar town, Eastern Ethiopia*.

### Populations

All the TB/HIV co-infected patients who registered in the public hospitals in Harar town were the source population. The study population was TB/HIV patients who systematically selected from those TB/HIV patients registered in the hospitals from 1st January 2012 to 31st December 2017. All the complete medical records of the TB/HIV co-infected patients who registered in the hospitals from1^st^ January 2012 to 31st December 2017 were included. The records of the patients with missing values on the variable’s interest were excluded. In addition, medical records of the transferred out patients were excluded since the TB treatment outcomes were unknown.

### Sample size determination and sampling technique

The sample size was calculated using a single population formula with 95% confidence interval (CI), a 5% margin of error and taking the proportion of successful treatment outcomes among TB/HIV co-infected patients from previous studies [16–19]. Then we took the largest sample size (324), which was based on a study conducted in Mizan Tepi of Ethiopia that found TB Treatment success rate of 30.3% [18]. After adding a 10% non-response rate for missing data, we found the final sample size to be 356.

This sample size was then allocated proportionally to the two selected public hospitals based on the number of registered TB cases in each hospital. Then in each selected hospital, the profiles of all TB/HIV co-infected patients were evaluated to check the fulfillment of the inclusion criteria. Finally, study subjects who met the inclusion criteria were selected from the TB registration Book through a systematic random sampling technique using calculated K^th^ value. We calculated the K-value by dividing expected patients during the study period to the number of samples and took every K^th^ patient in the registration book.

### Data collection tools and procedure

The data were collected through reviewing all the necessary documents (TB treatment registry, monthly cohort form, and follow up form) of the TB patients using a pre-tested structured data extraction format which was developed by considering the variables to be studied. The format contained all the important socio-demographic, baseline clinical and laboratory, and follow up data. Four public health officers who had training on comprehensive TB-HIV care and experience in collecting data in similar situations gathered the data. The data collectors were trained and the filled form was checked for completeness on a daily basis during the data collection. The whole process was supervised by the principal investigators.

### Data processing and analysis

The collected data were edited, cleaned, coded, entered and analyzed by using SPSS [Statistical package for social science] Version 21 for windows. Frequencies, proportions, and summary statistics were used to describe the study population in relation to socio-demographic and clinical characteristics. Binary logistic regression was calculated at 95% confidence intervals to evaluate the crude association between each exposure variable and outcome variable. Multi-collinearity was checked among independent variables like baseline CD4 count, WHO staging, functional status and history of opportunistic infections through variance inflation factor (VIF). No collinearity was found among the variables. Then to control the effect of confounding factors, a variable that had *P*-value ≤ 0.2 in the bivariate analysis was entered into the multivariate logistic regression model, as the independent variable and TB treatment outcome status being a dependent variable. Multivariate logistic regression analysis was employed to assess the independent association of each exposure variable with TB treatment outcome. *P*-value < 0.05 was considered statistically significant in the final model. Hosmer and Lemeshow test was used to check the assumption on the fitness of goodness of the final model and it was found fit.

### Ethical consideration

Ethical clearance was secured from the research review technical committee of Harar Health Science College. A legal permission letter was taken from Harar Health Science College to the selected public hospitals and official permission was obtained from the administration of the hospitals. Furthermore, before reviewing medical records of the TB/HIV co-infected patients, permission was obtained from the TB treatment unit heads.

The information obtained from the study was used only for the purpose of the study and is kept confidential. Since the data were collected through review of medical records, there is no harm to the patients and their relatives provided confidentiality is maintained. Moreover, no personal identifier was used on the data collection form. The recorded data were not accessed by a third person except the principal investigators.

### Definitions of treatment outcome

According to the standard definitions of the National Tuberculosis and Leprosy Control Program guideline (NTLCP) [20] and the WHO Definitions and reporting framework for tuberculosis 2013 revision [21], the following treatment outcome definitions were used:

- **✓ “Cured**: A pulmonary TB patient with bacteriologically confirmed TB at the beginning of treatment who was smear- or culture-negative in the last month of treatment and on at least one previous occasion.
- **✓ Treatment completed:** A TB patient who completed treatment without evidence of failure BUT with no record to show that sputum smear or culture results in the last month of treatment and on at least one previous occasion were negative, either because tests were not done or because results are unavailable.
- **✓ Treatment failure:** A TB patient whose sputum smear or culture is positive at 5 months or later during treatment. Or Patients found to harbor a multidrug-resistant (MDR) strain at any point of time during the treatment, whether they are smear-negative or -positive.
- **✓ Died**: A TB patient who dies for any reason before starting or during the course of treatment.
- **✓ Lost to follow-up:** A TB patient who has been on treatment for at least four weeks and whose treatment was interrupted for eight or more consecutive weeks
- **✓ Not evaluated**: A TB patient for whom no treatment outcome is assigned. This includes cases “**transferred out**” to another treatment unit as well as cases for whom the treatment outcome is unknown to the reporting unit”.

**In line with WHO criteria (21), treatment outcome is categorized into:**

- **✓ “Successful outcome**- if TB patients are cured (i.e., negative smear microscopy at the end of treatment and on at least one previous follow-up test) or completed treatment with resolution of symptoms”.
- **✓ “Poor outcome** – if treatment of TB patients resulted in treatment failure (i.e., remaining smear-positive after 5 months of treatment), lost to follow-up (i.e., patients who interrupted their treatment for two consecutive months or more after registration), or death”.

## Results

### Socio-demographic characteristics

A total of 349 TB/HIV co-infected patients under TB treatment were included. The charts three TB/HIV co-infected patients were excluded due to missing variables. Of the 349 patients, 188 (53.9%) were female and 284 (81.4%) were urban residents. Their ages ranged from 8 years to 65 years with a mean of 33.6 (SD ± 9.6) years); their baseline weight was from 20 to 100 Kg with a mean of 55. 0 (standard deviation (SD) ± 12.3) Kg; and their pretreatment body mass index (BMI) from 11.9 to 30 with a mean of 20.9 (SD ± 3.9). Most of the study participants (81.1%) were in the productive age group (16–45 years). Nearly half of the patients (47.6%) weighed more than 54Kg. The BMI of the 201 (57.6%) patients was within 18.5–24.9 (Table 1).

**Table 1:** Socio-demographic characteristics of TB/HIV co-infected patients at Public Hospitals of Harar town, Eastern Ethiopia, 2018

Variables	Frequency	Percent
Sex		
Male	161	46.1
Female	188	53.9
Age		
1–15 years	10	2.9
16–30 years	146	41.8
31–45 years	137	39.3
> 45 years	56	16.0
Place of residence		
Urban	284	81.4
Rural	65	18.6
Pretreatment Weight		
20–29 Kg	5	1.4
30–39 Kg	22	6.3
40–54 Kg	156	44.7
> 54 Kg	166	47.6
Pretreatment BMI (Kg/m^2^)		
< 18.5	98	28.1
18.5–24.9	201	57.6
≥ 25	50	14.3
Year of TB treatment initiated		
2012	41	11.7
2013	69	19.8
2014	70	20.1
2015	71	20.3
2016	64	18.3
2017	34	9.7

### Clinical characteristics

Majority (88.8%) of the study participants were new TB cases (95% CI: 85.7–92.0%). One hundred eleven of the study participants had smear-positive Pulmonary TB (31.8%), 119 had smear-negative Pulmonary TB (34.1%), and the remaining 119 had Extra-pulmonary TB (34.1%). About two-thirds of patients have been on working functional status (67%) and 128 (36.7%) were categorized to WHO stage 3 HIV disease during the initiation of their TB treatment. More than half (54.4%) of the patients had experienced opportunistic infections like pneumonia (37%), candidiasis (26%) and sexually transmitted infections (11%). The majority (84.8% with 95% CI: 80.8–88.5) of study participants were on CPT. The Cluster of Differentiation 4 (CD4) count of the patients ranged from 43 to 1058 cells/μl, with median (Inter Quartile Range (IQR)) of 298 (187.5–460.5) cells/μl; and only 75 (21.5%) patients had CD4 count more than 500 cells/μl (95% CI: 17.2–25.8). At the time of TB diagnosis, the patients have spent 1 to 15 years on ART with a median duration of 4.00 and IQR of 3–6 years. About two-thirds of the patients (64.8%) have spent less than 5-years on ART (Table 2).

**Table 2:** Clinical characteristics of TB/HIV co-infected patients at Public Hospitals of Harar town, Eastern Ethiopia, 2018

Variables	Frequency	Percent
TB clinic		
Jugal hospital	180	51.6
Hiwot Fana Specialized University Hospital	169	48.4
Category of patient		
New	310	88.8
Retreatment	39	11.2
Types of TB		
Smear positive PTB (SPP TB)	111	31.8
Extra-pulmonary TB (EP TB)	119	34.1
Smear negative PTB (SNP TB)	119	34.1
Functional status ^a^		
Working	234	67.0
Ambulatory	106	30.4
Bedridden	9	2.6
WHO staging		
stage 1	79	22.6
stage 2	102	29.2
stage 3	128	36.7
stage 4	40	11.5
History of opportunistic infection^c^		
No	159	45.6
Yes	190	54.4
Types of Opportunistic infections (*n* = 190)		
Pneumonia	71	37.4
Candidiasis	49	25.8
Sexually transmitted infections,	21	11.1
Intestinal parasites,	19	10.0
Urinary tract infections	17	8.9
Others	13	6.8
CPT initiated		
Yes	296	84.8
No	53	15.2
Baseline CD4 count		
< 200 cells/μL	102	29.2
201–499 cells/μL	172	49.3
> 500 cells/μL	75	21.5
Duration on ART		
Less than 5 years	226	64.8
More than 5 Years	123	35.2
Positive smear result (*n* = 230)		
After 2 month	26	11.3
After 5 month	13	5.7
After 7 month	13	5.7

^a^ **Working**: “able to perform usual work in or out of the house, harvest, go to school or, for children, normal activities or playing”, **Ambulatory**: “able to perform activities of daily living but not able to work or play” and **Bedridden**: “not able to perform activities of daily living” (22)

^b^ including co-infection

### Treatment outcome of TB- HIV co-infected patients on anti-TB therapy

**Fig. 1:**
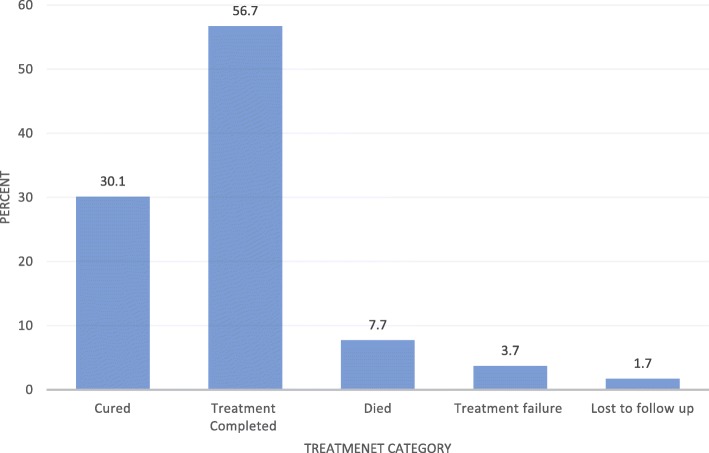
TB treatment outcome of TB-HIV co-infected patients attending public hospitals of Harar town, Eastern Ethiopia, 2018

Among the TB-HIV co-infected patients, 105 (30.1% with 95% CI: 25.5–34.9) were cured, 198 (56.7% with 95% CI: 51.6–61.6) had completed their treatment, 27 (7.7% with 95% CI: 4.9–10.6) died, 6 (1.7% with 95% CI: 0.6–3.2) were lost to follow up from their treatment and the remaining 13 (3.7% with 95% CI: 1.7–5.7) were treatment failure **(**Fig. 1). Overall, 303 (86.8%) (95% CI: 83.1–90.3) of the TB-HIV co-infected patients had successful TB treatment outcome whereas, the remaining 46 (13.2%) (95% CI: 9.7–16.9) patients had unsuccessful TB treatment outcome.

### Factors associated with successful TB treatment outcome

The association between successful TB treatment outcome and the socio-demographic and clinical characteristics of the study participants was assessed through bivariate and multivariate analyses.

The treatment success rate was similar between male (87.6%) and female (86.2%) patients. The urban study participants had slightly higher (87.7%) successful TB treatment outcome than the rural ones (83.1%). TB treatment success rate was 82.7, 87.6 and 92% in the patients with baseline BMI less than 18.5, 18.5 to 24.9, and greater than 25, respectively. However, the increment in the success rate was not statistically significant (*p*. value =0.236). The bivariate analysis showed that successful TB treatment outcome was not statistically associated with sex, age, place of residence, baseline BMI, and year of treatment of the TB/HIV co-infected patients (Table 3).

**Table 3:** Bivariate analysis of TB Treatment outcome with socio-demographic of TB/HIV co-infected patients attended Public hospitals of Harar town, Eastern Ethiopia, 2018

Characteristics	Treatment outcome		*P*- value	Crude Odds Ratio (COR)(95%CI)
	Successful Treatment	Unsuccessful Treatment		
Sex				
Male	141 (87.6%)	20 (12.4%)	0.699	1.00
Female	162 (86.2%)	26 (13.8%)		0.88 (0.44–1.57)
Age group				
1–15 years	9 (90.0%)	1 (10.0%)	0.741	1.43 (0.17–11.89)
16–30 years	126 (86.3%)	20 (13.7%)		1.00
31–45 years	122 (89.1%)	15 (10.9%)	0.483	1.29 (0.63–2.64)
> 45 years	46 (82.1%)	10 (17.9%)	0.458	0.73 (0.32–1.68)
Place of residence				
Urban	249 (87.7%)	35 (12.3%)	0.325	1.00
Rural	54 (83.1%)	11 (16.9%)		0.69 (0.33–1.44)
Pretreatment weight				
20–29 Kg	4 (80.0%)	1 (20.0%)	0.529	0.49 (0.05–4.59)
30–39 Kg	16 (72.7%)	6 (27.3%)	**0.037**	**0.32 (0.11–0.93)**
40–54 Kg	135 (86.5%)	21 (13.5%)	0.473	0.78 (0.40–1.53)
> 54 Kg	148 (89.2%)	18 (10.8%)		1.00
Pretreatment BMI (kg/m^2^)				
< 18.5	81 (82.7%)	17 (17.3%)		1.00
18.5–24.9	176 (87.6%)	25 (12.4%)	0.251	1.48 (0.76–2.89)
≥ 25	46 (92.0%)	4 (8.0%)	0.132	2.41 (0.77–7.61)
Year of TB treatment initiated				
2012	36 (87.8%)	5 (12.2%)		1.00
2013	61 (88.4%)	8 (11.6%)	0.925	1.06 (0.32–3.48)
2014	60 (85.7%)	10 (14.3%)	0.756	0.83 (0.26–2.63)
2015	62 (87.3%)	9 (12.7%)	0.941	0.96 (0.30–3.08)
2016	57 (89.1%)	7 (10.9%)	0.843	1.13 (0.33–3.84)
2017	27 (79.4%)	7 (20.6%)	0.328	0.54 (0.15–1.87)

Significant values are set in bold

On the other hand, types of TB, functional status, WHO staging, history of opportunistic infection, and CPT initiated showed statistically significant association with TB treatment outcome among the TB/HIV co-infected patients. Those on the retreatment category had a higher unsuccessful treatment rate (23.1%) than the new TB cases (11.9%). In this study, smear-positive PTB patients had higher unsuccessful treatment outcomes (18.9%) than extrapulmonary TB (14.3%) and smear-negative PTB (6.7%) cases and this difference was statistically significant (p. value = 0.028). More than half of the patients under the category of bedridden functional status had unsuccessful treatment outcome but only 11.5% of the patients with working functional status had unsuccessful treatment (P. value = 0.005). Similarly, about 30% of the TB/HIV co-infected patients with WHO stage 4 diseases had an unsuccessful treatment outcome whereas 8.9% of the patients with stage 1 disease had unsuccessful treatment. (P. value = 0.003). A higher proportion of the TB/HIV co-infected patients with a history of opportunistic infection (18.9%) had unsuccessful TB treatment outcomes than those who had no history of opportunistic infection (6.3%) (Table 4**)**.

**Table 4:** Bivariate analysis of TB Treatment outcome with Clinical characteristics of TB/HIV co-infected patients attended Public hospitals of Harar town, Eastern Ethiopia, 2018

Characteristics	Treatment outcome		*P*- value	COR (95%CI)
	Successful Treatment	Unsuccessful Treatment		
Patient category				
New cases	273 (88.1%)	37 (11.9%)	0.058	1.00
Re treatment	30 (76.9%)	9 (23.1%)		0.45 (0.20–1.03)
Types of TB				
SPP TB	90 (81.1%)	21 (18.9%)		1.00
EP TB	102 (85.7%)	17 (14.3%)	0.346	1.40 (0.70–2.82)
SNP TB	111 (93.3%)	8 (6.7%)	0.007	3.24 (1.37–7.65)
Functional status				
Working	207 (88.5%)	27 (11.5%)		1.00
Ambulatory	92 (86.8%)	14 (13.2%)	0.662	0.86 (0.43–1.71)
Bedridden	4 (44.4%)	5 (55.6%)	0.001	0.10 (0.03–0.41)
WHO staging				
stage 1	72 (91.1%)	7 (8.9%)		1.00
stage 2	94 (92.2%)	8 (7.8%)	0.806	1.14 (0.39–3.30)
stage 3	109 (85.2%)	19 (14.8%)	0.212	0.56 (0.22–1.39)
stage 4	28 (70.0%)	12 (30.0%)	0.005	0.23 (0.08–0.64)
History of opportunistic infection				
No	149 (93.7%)	10 (6.3%)	0.001	1.00
Yes	154 (81.1%)	36 (18.9%)		0.29 (0.14–0.60)
CPT initiated				
Yes	264 (89.2%)	32 (10.8%)	0.003	1.00
No	39 (73.6%)	14 (26.4%)		0.34 (0.17–0.69)
CD4 count				
< 200 cells/μL	83 (81.4%)	19 (18.6%)		1.00
201–499 cells/μL	155 (90.1%)	17 (9.9%)	0.041	2.09 (1.03–4.23)
± 500 cells/μL	65 (86.7%)	10 (13.3%)	0.349	1.49 (0.65–3.42)
Duration on ART				
< 5 years	199 (88.1%)	27 (11.9%)	0.357	1.00
> 5 Years	104 (84.6%)	19 (15.4%)		0.74 (0.39–1.39)

Multivariate logistic regression revealed that patient category, types of TB, WHO staging, history of opportunistic infection, and CPT initiated were significantly associated with TB treatment outcome among the TB/HIV co-infected patients. Patients on the retreatment category had 2.91 times (AOR = 2.91, 95% CI: 1.17–7.28) higher odds of unsuccessful treatment outcome compared with the new cases. Those patients with extrapulmonary TB and smear-negative pulmonary TB had 76.5% (AOR = 0.235, 95% CI: 0.090–0.617) and 65.7% (AOR = 0.343, 95% CI: 0.129–0.911) lower odds of having unsuccessful TB treatment outcome, respectively, than the patients with smear-positive pulmonary TB.

The odds of having unsuccessful TB treatment outcome was 4.46 (AOR =4.46, 95% CI: 1.24–16.02), 5.94 (AOR = 5.94, 95% CI: 1.87–18.85), and 3.01 (AOR = 3.01, 95% CI: 1.15–7.91) times higher among the patients in clinical stage 2, clinical stage 3, and clinical stage 4 HIV disease respectively than those in clinical stage 1 disease. The patients with opportunistic infection history had 3.68 (AOR = 3.68, 95% CI: 1.62–8.33) times higher odds of having unsuccessful TB treatment outcomes than those without opportunistic infection history. Similarly, the odds of unsuccessful TB treatment outcome was 3.54 (AOR = 3.54, 95% CI: 1.59–7.89) times higher among the patients who were not put on CPT compared with patients on CPT (Table 5).

**Table 5:** Multivariate analysis of TB Treatment outcome with socio-demographic and Clinical characteristics of TB/HIV co-infected patients attended Public hospitals of Harar town, Eastern Ethiopia, 2018

Characteristics	Treatment outcome		*P*- value	Adjusted Odds Ratio (AOR) (95% CI)
	Successful Treatment	Unsuccessful treatment		
Pretreatment Weight				
20–29 Kg	4 (80.0%)	1 (20.0%)	0. 843	0.92 (0.38–2.19)
30–39 Kg	16 (72.7%)	6 (27.3%)	0.244	0.22 (0.02–2.79)
40–54 Kg	135 (86.5%)	21 (13.5%)	0.150	0.40 (0.12–1.39)
> 54 Kg	148 (89.2%)	18 (10.8%)		1.00
Pretreatment BMI (kg/m^2^)				
< 18.5	81 (82.7%)	17 (17.3%)		1.00
18.5–24.9	176 (87.6%)	25 (12.4%)	0.834	0.85 (0.18–4.01)
≥ 25	46 (92.0%)	4 (8.0%)	0.497	0.64 (0.17–2.34)
Patient category				
New cases	273 (88.1%)	37 (11.9%)	0.022	1.00
Re treatment	30 (76.9%)	9 (23.1%)		**2.91 (1.17–7.28)**
Types of TB				
SPP TB	90 (81.1%)	21 (18.9%)		1.00
EP TB	102 (85.7%)	17 (14.3%)	**0.003**	**0.23 (0.09–0.62)**
SNP TB	111 (93.3%)	8 (6.7%)	**0.032**	**0.34 (0.13–0.91)**
WHO staging				
stage 1	72 (91.1%)	7 (8.9%)		1.00
stage 2	94 (92.2%)	8 (7.8%)	**0.022**	**4.46 (1.24–16.02)**
stage 3	109 (85.2%)	19 (14.8%)	**0.003**	**5.94 (1.87–18.85)**
stage 4	28 (70.0%)	12 (30.0%)	**0.025**	**3.01 (1.15–7.91)**
History of opportunistic infection				
No	149 (93.7%)	10 (6.3%)	0.002	1.00
Yes	154 (81.1%)	36 (18.9%)		**3.68 (1.62–8.33)**
CPT initiated				
Yes	264 (89.2%)	32 (10.8%)	**0.002**	1.00
No	39 (73.6%)	14 (26.4%)		**3.54 (1.59–7.89)**
CD4 count				
< 200 cells/μL	83 (81.4%)	19 (18.6%)		1.00
201–499 cells/μL	155 (90.1%)	17 (9.9%)	0.451	1.50 (0.52–4.33)
≥ 500 cells/μL	65 (86.7%)	10 (13.3%)	0.433	1.47 (0.56–3.82)

Significant values are set in bold

## Discussion

Tuberculosis and HIV/AIDS constitute the main burden of infectious disease in resource-limited countries [1, 2]. Determining the TB treatment outcome among TB-HIV co-infected patients in different settings may provide evidence for evaluating the performance of the TB control program in the country and forward future direction.

According to WHO 2017 Global Tuberculosis Report, the global treatment success rate for HIV-associated TB cases among the 2015 cohort was 78% and in the WHO Africa region, it was 80% [23]. In this study, the overall TB treatment success rate among the TB-HIV co-infected patients was 86.8%, which is similar to the results reported from studies carried out in rural South Africa (82.2%) [24], Ahmedabad (84.2%) [25] and Addis Ababa, Ethiopia (88.2%) [26]. Our finding is lower than that of the Chandigarh study which reported a treatment success rate of 93.1% [27]. On the other hand, TB treatment success rate of our study is higher than several studies conducted in Gondar (22.6%) [28], Mizan Aman (28.5%) [17], Mizan Tepi (30.3%) [18], Malaysia (53.4%) [29], Brazil (55%) [30], Western Ethiopia (60.7%) [31], Cameroon (60.8%) [32], Iran (64%) [33], Ebonyi State Nigeria (65.8%) [34], Karnataka India (66.1%) [35], Tigray (70.8%) [16], Viet Nam (73%) [36], Arsi Negele (73%) [37], Yavatmal India (75%) [38], South India (75%) [39] and Free State province of South Africa (75.5%) [40].

This observed variation might be due to the difference in the quality of service in the TB/HIV clinic; proper counseling, health education, and appropriate follow up by the clinician. Another possible explanation might be the inclusion of transferred out patients in the final analysis by some of the previous studies. The proportion of transferred out TB/HIV co-infected patients were ranging from 3.8% in the Ebonyi State of Nigeria [34] to 64.2% in Mizan Aman of Ethiopia [17]. But, in our study, we did not include transferred patients because their records were not available and their treatment final outcomes were unknown. Furthermore, the high success rate observed in our study could be due to the initiation of ART for all co-infected patients. As it has been reported in previous studies [16, 24, 29, 31–33, 35, 36, 39], patients who had ART initiated were found to have high success rate compared with their counterpart. For instance, studies conducted in Tigray Ethiopia [16] and Malaysian [29] stated that TB/HIV co-infected patients who were not receiving ART were 3.42 and 5.10 times more likely to have unsuccessful TB treatment outcomes than those who received ART, respectively.

TB/HIV co-infected patients have increased mortality due to rapid disease progression, late diagnosis and other opportunistic infections [4, 6]. In the current study, the death rate among the TB/HIV co-infected patients during TB treatment was 7.7%. Also, similar death rates were seen in study carried out in Mizan Aman (6%) [17], Aurangabad city, India (8.2%) [41], Addis Ababa, Ethiopia (8.3%) [26], South India (9%) [39], WHO African Region (9%) [23], Gondar (9.6%) [28], Viet Nam (10%) [36] and South Africa (10.5%) [24]. The death rate in our study was lower than the rate reported in deferent part of Ethiopia which reported a death rate ranging from 12.8 to 20.2% [16, 18, 31, 37, 42–44], Cameroon 29.4% [32], Karnataka India 15.7% [45], Yavatimal India 16% [38], Malaysia 21% [29], Ebonyi State Nigeria 19% [34], Free State province South Africa 17.4% [40], Iran 18.9% [33]. Moreover, the WHO reported that TB associated death rate among HIV-positive TB patients was 11% [23]. Nonetheless, smaller death rates were reported from Chandigarh India 1.14% [27], Ahmedabad 4.17% [25] and Brazil 4.3% [30]. The lower death rate in our study could be explained by the fact that all our study participants had been on ART. ART is protective against mortality treatment outcomes during TB treatment. as demonstrated by previously conducted in Tigray [16], Karnataka India [35], south India [39] and North West Ethiopia [43].

Lost for follow up from TB treatment program is a major public health problem that can be associated with adverse drug reactions, social stigma and lack of awareness of the disease [17]. The proportion of lost for follow up found in this study (1.7%) is comparable Mizan Aman 1.3% [17], Tigray 2% [16] and Gondar 2.2% [28] studies. This lost for follow up of TB/HIV co-infected patients is lower than many studies conducted in Ethiopia [18, 31], Africa [24, 32, 34, 40] and Asia [29, 35, 36, 39, 41]. The possible explanation might be a good implementation of the DOT program. In addition, an increased patient awareness, and treatment adherence, and service accessibility might have contributed to this smaller proportion of lost for follow up in our settings.

The management of TB and HIV co-infected individuals is challenging because of the high pill burden, increased the adverse effect and drug-drug interaction [6]. In addition, several factors play a significant role in determining the TB treatment outcome among TB/HIV co-infected patients. In this regard, our study revealed that patient category, types of TB, WHO staging, history of opportunistic infection and CPT initiation significantly associated with TB treatment outcome.

The TB/HIV co-infected patients on the retreatment category had a higher chance of unsuccessful treatment outcome than the new patients. A similar result was reported from South India [39], Brazil [30], Yavatmal India [38] and Viet Nam [36] studies. The poor treatment outcome observed in the retreatment category could be due to drug resistance because of re-exposure to the drugs [30].

Type of TB infection was identified by different previous studies [18, 26, 29, 30, 37–39, 43, 44] as a determinant of TB treatment outcome. In our study, the chance of unsuccessful treatment was lower among the TB/HIV co-infected patients with extrapulmonary TB and smear-negative pulmonary TB compared with the smear-positive pulmonary TB. A similar, finding was reported by a study conducted in Addis Ababa Ethiopia [26], South India [39] and Yavatmal India [38].

In contrary to this, a study conducted in Mizan Tepi [18] demonstrated TB/HIV co-infected patients with Smear positive PTB had a higher chance of successful TB treatment outcome. In addition, studies conducted in many places [29, 37, 43, 44] reported that TB/HIV co-infection patients with extrapulmonary TB had a higher chance of mortality during TB treatment than pulmonary TB patients. In general, a high successful treatment outcome is expected among patients with smear-positive PTB compared with smear-negative PTB and extrapulmonary TB. However, the higher successful treatment outcome observed in our study among smear-negative PTB and extrapulmonary TB patients might be due to the close supervision and attention given by health care provides to these groups of patients. This close supervision might be associated with considering these patients are at high risk for unsuccessful treatment outcomes.

In the present study, similar to studies conducted elsewhere (MizanTepi [18] and Tigray [16]), the chance of unsuccessful TB treatment outcome was higher among the TB/HIV co-infected patients in stage 2, stage 3 and stage 4 HIV diseases than those in stage 1 disease. This might be due to the development of different opportunistic infections among the patients with advanced stages of HIV which can directly affect TB treatment outcomes.

The association between opportunistic infections and TB treatment outcomes was also demonstrated by our study. Those TB/HIV co-infected patients with opportunistic infection history had a higher chance of unsuccessful TB treatment outcome than the patients without opportunistic infection history. This is in line with a finding reported in Cameroon [32]. The possible reason could be poor adherence to anti TB drugs due to the high pill burden and associated drugs adverse effect.

Similarly, the chance of unsuccessful TB treatment outcome was higher among the TB/HIV co-infected patients who did not take CPT compared with those who took the prophylaxis. Studies conducted in North West Ethiopia [43], Cameroon [32], South Africa Free State [40], Yavatmal India [38] and Viet Nam [36] also reported similar findings. The administration of CPT can reduce the chance of co-morbidities such as pneumonia with pneumocystis, toxoplasmosis and other bacterial infections, which may then be related to a higher rate of cure and treatment completion and a lower rate of deaths.

According to the Ethiopia National Guidelines, CPT should be provided for all HIV positive TB patients as one component of the national TB/HIV collaborative activity [6]. In spite of this, only 84.8% of TB/HIV co-infected patients were on CPT. This finding was consistent with studies from western Ethiopia (80%) [31], Yavatmal India (80%) [38], Gondar (88.9%) [19] and Addis Ababa Ethiopia (77%) [26]. However, this figure was higher than a report from Ebonyi State Nigeria (55.3%) [34] and Free State province South Africa (42.1%) [40]. Hence, health care providers should apply every effort to make sure that all HIV patients took CPT.

### Limitation of the study

The major limitation of this study is related to the use of retrospective secondary data. Some important variables which might have impact on treatment outcome of TB/HIV co-infected patients, like socioeconomic characteristics (income, family size, educational status, living condition, social support, distance to the health facility), treatment and disease-related variables (adherence level, viral load, drug resistance), as well as behavioral factors (knowledge and attitude about the diseases, alcohol abuse, cigarette smoking, illicit drug use) were not recorded. Moreover, some patients were transferred to other health facilities where it is difficult to track what happened thereafter. Exclusion of medical records of a patient who were transferred out and/or found to be incomplete may have also slightly affected our results. This study also involved only patients in public hospitals. Its result may not be applicable to the patients in the private setting.

## Conclusion

The overall TB treatment success rate among the TB-HIV co-infected patients in this study was higher compared with many previous studies. TB/HIV patients with a history of previous TB treatment, smear-positive pulmonary TB, advanced HIV stage, history of opportunistic infection and no CPT initiated were at a high risk of getting poor treatment outcomes.

Therefore, TB treatment facilities should give special attention to those TB-HIV co-infected patients with a higher risk of unsuccessful TB treatment outcome.

## Data Availability

The datasets of the study are available on reasonable request from the corresponding author.

## Abbreviations

AOR: Adjusted Odds Ratio
ART: Antiretroviral Therapy
BMI: Body mass index
CD4: Cluster of Differentiation 4
CI: Confidence interval
COR: Crude Odds Ratio ()
CPT: Co-trimoxazole prophylaxis
DOT: Directly Observed Therapy
EP TB: Extra-pulmonary TB
HFSUH: Hiwot Fana Specialized University Hospital
HHSC: Harar Health Science College
HIV: Human Immunodeficiency Virus
IPT: Isoniazid preventive therapy
IQR: Inter Quartile Range
MDR: Multi-drug Resistant
PTB: Pulmonary TB
SD: Standard deviation
SNP TB: Smear negative PTB
SPP TB: Smear positive PTB
SPSS: Statistical package for social science
TB: Tuberculosis
TB/HIV: Tuberculosis/ Human Immunodeficiency Virus
WHO: World health organization
